# Psychosocial interventions to improve tuberculosis preventive treatment uptake and psychosocial outcomes: a systematic review

**DOI:** 10.1038/s41533-025-00449-3

**Published:** 2025-09-29

**Authors:** Ida A. A. Parwitha, Vania D. Djunaidy, Sofa D. Alfian, Hari Setyowibowo, Ivan S. Pradipta

**Affiliations:** 1 Doctoral Program of Pharmacy, Faculty of Pharmacy, Universitas Padjadjaran, Bandung, Indonesia; 2 Department of Clinical and Community Pharmacy, Faculty of Pharmacy, Widya Mandala Surabaya Catholic University, Surabaya, Indonesia; 3 Department of Pharmacology and Clinical Pharmacy, Faculty of Pharmacy, Universitas Padjadjaran, Bandung, Indonesia; 4 Drug Utilization and Pharmacoepidemiology Research Group, Center of Excellence in Higher Education for Pharmaceutical Care Innovation, Universitas Padjadjaran, Sumedang, Indonesia; 5 Department of Psychology, Faculty of Psychology, Universitas Padjadjaran, Bandung, Indonesia

**Keywords:** Diseases, Health care, Medical research, Psychology, Psychology

## Abstract

Despite its importance in global TB elimination, tuberculosis preventive treatment (TPT) remains underutilized. Psychosocial barriers significantly contribute to this issue. This systematic review aims primarily to synthesize psychosocial interventions to improve the initiation of TPT. We analyzed psychosocial outcomes as secondary objectives when relevant data were available. This review included studies indexed in PubMed, Scopus, and PsycInfo until August 25, 2025. Original studies addressing psychosocial interventions for people with latent tuberculosis infection (LTBI) indicated for TPT were included in this review. The risk of bias was assessed via the Crowe Critical Appraisal Tool (CCAT). A narrative synthesis summarized the characteristics of interventions, including the format of delivery, settings, intervention providers, psychosocial content, duration, and outcomes. Among the 1725 identified studies, nine (14,428 participants) met the inclusion criteria. The CCAT classification was moderate to high quality, with strengths in clearly articulated study rationales but weaknesses in study design. Most studies were from upper-middle-income countries with a high burden of TB; none were from Asia. Health education is a core component, often incorporating culturally adapted survivor testimonials to reduce stigma and increase motivation. Interventions were mostly community-based and led by multidisciplinary healthcare professionals and community workers. TPT initiation improved in all included studies, with risk differences ranging from 10–52%. This review emphasizes the potential of psychosocial interventions in supporting behavior change and increasing TPT initiation. Methodological limitations and a lack of research in high-burden Asian contexts restrict the current evidence. Future studies should focus on developing rigorous, contextually appropriate strategies for scalable psychosocial interventions that are effective and sustainable.

## Introduction

Tuberculosis (TB) continues to be a significant worldwide public health concern, with approximately 10.8 million cases and 1.3 million deaths^1^. This situation requires immediate attention and action to prevent further spread and mortality. Despite widespread global commitment and notable progress, reaching the End TB Strategy goal of an 80% reduction in TB incidence by 2030 continues to be a significant challenge^1^. One major obstacle to achieving the end-TB goal is that 5–10% of people with latent tuberculosis infection (LTBI) may progress to TB disease, perpetuating the transmission cycle^2,3^. Administering tuberculosis preventive treatment (TPT) to high-risk groups, such as household contacts of TB patients or people living with HIV (PLHIV), is a key component of the End TB strategy^4^.

TPT is widely acknowledged as a crucial component of TB prevention strategies. Proper use of TPT can lower the risk of TB reactivation by 60–90%, offering a promising approach that could change how TB disease is prevented^5,6^. Despite its importance, TPT uptake is still low worldwide^1^. In response, the second United Nations High-Level Meeting on TB in 2023 set an ambitious target of providing TPT to 90% of high-risk individuals by 2027, aiming to reach 45 million people globally. This includes 30 million household contacts and 15 million PLHIV. However, by 2023, global coverage was still limited, with only 21% of household contacts and 56% of PLHIV having received TPT^1^.

Many people with LTBI decline to start preventive therapy, although universal health coverage covers it. Even if they initially agree, TPT initiation rates decline due to complex barriers. Evidence suggests that psychosocial determinants play a significant role in patients' decisions to initiate TPT^7–10^. Despite sufficient knowledge and awareness of the benefits of TPT, persistent TB-related stigma continuously reduces the likelihood of starting TPT^8–13^. The interaction between psychosocial barriers and TPT acceptance suggests that an educational approach alone may be inadequate without targeted interventions^8,13^. Hence, interventions addressing psychosocial barriers are critical.

Providing psychosocial interventions, including economic, nutritional, and psychological support, is crucial in the people-centered approach outlined in the WHO's End TB Strategy. These interventions are aimed not only at improving individual health outcomes but also at addressing the social and economic factors that contribute to the transmission and progression of TB^14^. Most studies focus on the implementation phase, even though many eligible individuals do not start treatment and are lost to follow-up early^15^. Psychosocial interventions that increase TPT uptake are underexplored^15,16^. Therefore, this systematic review primarily synthesizes the variety of psychosocial interventions and their effects on the initiation of TPT. In addition, we analyzed psychosocial outcomes (e.g., stigma, knowledge, and perceived support) as secondary objectives when relevant data were available. The findings of this review will guide the development of scalable psychosocial interventions that facilitate progress toward the elimination of TB.

## Methods

The reporting of this systematic review was conducted following the PRISMA (Preferred Reporting Items for Systematic Reviews and Meta-Analyses) guidelines^17^. The protocol is registered on PROSPERO (CRD420251081568) and can be accessed online.

### Eligibility criteria

This systematic review examined existing evidence on psychosocial interventions that facilitate the initiation of TPT in people with LTBI. The target population included people eligible for TPT, such as household contacts, PLHIV, and broader high-risk community groups. Psychosocial interventions are defined as structured activities addressing non-biomedical factors that may impede people from adhering to treatment plans and are provided in conjunction with biomedical care^18^. The scope of psychosocial interventions in this review aligned with the psychosocial support (PSS) pyramid, a stepped framework that addresses the psychological, social, and economic determinants of care^19^. The psychosocial components encompassed four approaches: (i) education and counseling; (ii) emotional support, peer or family engagement, and stigma reduction activities; (iii) social protection or livelihood assistance (e.g., food, transport, or cash support); and (iv) referral to or integration with specialist services, such as mental health or substance-use care. These interventions were delivered by healthcare professionals (HCPs) or community health workers (CHWs). Healthcare professionals, including physicians, nurses, psychologists, and pharmacists, are trained personnel with formal qualifications recognized by higher education institutions^20^. Community health workers include village health workers, nongovernmental organizations (NGOs), and trained volunteers. This category usually requires informal training and supervision recognized by health and social service authorities^20^. We considered articles reporting key outcomes: (1) TPT initiation assessed through pre-post study designs or comparisons between intervention and control groups, and/or (2) psychosocial outcomes, such as knowledge, perceived risk benefits, perceived family support, and stigma. These outcomes were eligible for inclusion, whether they had been reported as primary or secondary endpoints in the original studies. This review included original studies, such as randomized controlled trials (RCTs), nonrandomized comparative studies, and observational studies. Qualitative studies with narrative data on barriers to TPT initiation were excluded; mixed-methods studies were included if they had relevant quantitative data. Systematic reviews were excluded; however, their reference lists were screened for additional relevant primary studies that may have been missed during the initial database search.

### Search strategy

A systematic search was conducted in PubMed, Scopus, and PsycInfo until August 25, 2025, to identify peer-reviewed articles. There were no restrictions on publication date or language. The complete search strategy is detailed in Supplementary Materials Table 1.

### Study and data selection

We used the Rayyan QCRI for systematic screening of records^21^. The screening process involved three stages: (1) screening titles and abstracts; (2) screening the full text with two independent reviewers; and (3) final screening during data extraction. One reviewer (IAAP) initially screened all titles and abstracts from the databases. To minimize selection bias, a random 10% sample of the title and abstract records was independently reviewed by a second reviewer (VDD). Two reviewers (IAAP and VDD) independently screened the full-text articles, resolving discrepancies through discussion, with a third reviewer (SDA, HS, ISP) involved as needed if consensus was not achieved. Finally, one reviewer (IAAP) conducted data extraction, which was then cross-checked by three other reviewers (SDA, ISP, and HS). The process of selecting studies was documented using a PRISMA flow diagram, and the reasons for excluding articles were systematically recorded^17,22^. The information collected included the following: (1) study characteristics (study authors, publication years, country, study design, target population, and settings); (2) psychosocial intervention model (type of psychosocial intervention, delivery format, content details, and duration); and (3) outcomes (risk differences and psychosocial outcomes).

### Quality assessment

Two researchers (IAAP and VDD) thoroughly assessed the full-text articles and compared their findings. To minimize researcher bias, each assessment was cross-verified. Discrepancies were resolved through consultation with a third reviewer (ISP, HS, SDA). Study quality was evaluated via the Crowe Critical Appraisal Tool (CCAT), version 1.4^23^. The CCAT was chosen for its ability to evaluate research, especially in assessing studies with varied designs and methods, making it especially suitable for complex psychosocial interventions^24–26^. In line with published guidance and previous systematic reviews, including the approach used by Nuttall et al., a CCAT score between 75 and 100% is deemed high quality, scores between 50 and 74% are considered moderate, and those below 49% are classified as low quality^23,27^.

### Data analysis

We performed a narrative synthesis following the methodological guidance developed by Popay et al., which is a robust method for systematic reviews involving different interventions^28^. This method was chosen to address the considerable heterogeneity across the included studies regarding psychosocial intervention components, study designs, target populations, and measurement outcomes. Performing a meta-analysis is inappropriate when substantial heterogeneity exists, as it can lead to misleading conclusions^29^. To ensure comparability and provide a clear foundation for determining the most promising psychosocial interventions, we calculated the risk difference (RD) for TPT initiation across all included studies. This method enabled us to standardize the reporting of effect sizes across various study types, facilitating direct comparisons of intervention impacts.

In addition to narrative synthesis, the analysis employed theory-driven, interpretive mapping to clarify the mechanisms underlying behavioral changes. Psychosocial intervention components were classified by target population and coded for their active ingredients using the Behavior Change Technique Taxonomy version 1 (BCT)^30^. We linked the psychosocial components to hypothesized psychosocial determinants (e.g., improved knowledge, perceived risk-benefit, stigma, perceived support or peer support, reduced access barriers, strengthened self-efficacy, and psychological readiness). The determinants were conceptualized as mechanistic pathways of behavior change and mapped to the COM-B domains (Capability, Opportunity, and Motivation)^31^. The summarized conceptual framework distinguishes empirically measured pathways from theory-informed but unproven mechanisms. This framework offers a structured and theory-driven lens for interpreting how psychosocial interventions may influence TPT initiation.

## Results

### Selection and inclusion of studies

**Fig. 1:**
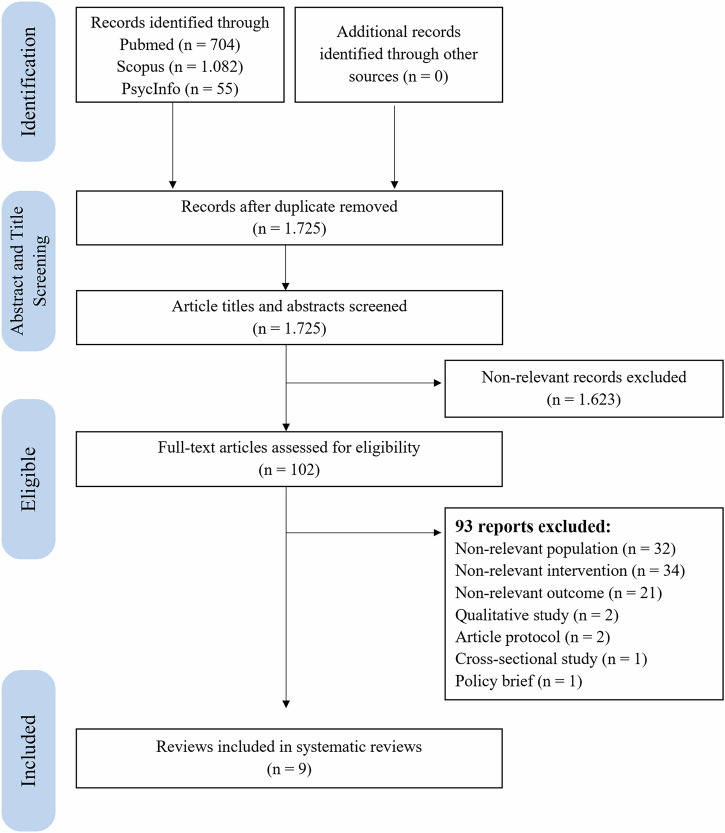
Flow diagram of the study selection.

The study selection process adhered to the PRISMA 2020 guidelines, as illustrated in Fig. 1. The initial systematic search yielded 704 records from PubMed, 1082 from Scopus, and 55 from PsycInfo, resulting in a total of 1841 articles. After removing 116 duplicates, 1725 records were available for screening. Title and abstract screening were conducted by one reviewer (IAAP), with a random 10% sample independently reviewed by a second reviewer (VDD), resulting in 100% agreement. Thus, 1623 records were excluded because they were irrelevant to either TPT initiation or psychosocial interventions, leaving 102 articles for full-text review. Of these, 93 articles were excluded for the following reasons: 32 did not meet the population criteria (e.g., they focused on individuals with TB disease or drug-resistant TB); 21 reported outcomes unrelated to TPT (e.g., uptake of tuberculin screening or IGRA tests); 34 articles involved interventions that did not align with the operational definition of psychosocial intervention; 2 articles were solely qualitative assessments of barriers in TPT uptake without evaluating interventions; 2 articles were study protocols; 1 article was a cross-sectional study on the TPT uptake rate; and 1 was a policy brief. Finally, nine studies met all the inclusion criteria and were included in further analysis.

### Characteristics of the included studies

The included studies encompass a diverse range of geographic areas and intervention activities, underscoring the widespread recognition of psychosocial interventions as determinants in initiating TPT (Table 1). Among the nine studies, seven (77.8%) were conducted in high TB burden (HTB) countries, including three studies in lower-middle-income (LMI) countries (Ethiopia^32^, Lesotho^33^, and El Salvador^34^) and four in upper-middle-income (UMI) countries (Peru^35–37^, Namibia^38^). The remaining two studies (22.2%) were conducted in high-income countries (HIC) with low TB burdens (LTB) (Canada^39^ and the Netherlands^40^). In terms of study design, five studies^34,35,37–39^ employed an uncontrolled pre-post design, one study^32^ implemented comparative pre-post designs with concurrent controls, two studies^33,36^ conducted randomized controlled trials (RCTs), and one study^40^ employed a mixed-methods implementation approach.

**Table 1:** Characteristics and outcomes of the included studies.

No	Authors	Settings and Participants	Study design and control group	Interventions and psychosocial contents	Format delivery and duration	Outcomes	Quality of Evidence
1.	Rocha et al.^35^	Settings: Community, Peru (UMI, HTB). Participants: Household TB-contacts aged < 19 years.	Study design: Uncontrolled pre-post design. Control group: Pre-intervention data were collected from 2003 to 2007 in the same communities.	Interventions: An integrated model of socioeconomic interventions through regular home visits to every household affected by TB. The core components were: (1) Psychological counseling; (2) Community-mobilization workshops; (3) Food and cash transfers of ± $ 160 per household; and (4) Income-generation support. Contents: a. Education content: TB diagnosis, preventive treatment, and treatment adherence. b. Psychological support: management of depression and substance use, reduction of TB-related stigma, and addressing other psychosocial issues. c. Financial education: Health insurance registration and skills training for income generation.	Verbal presentation; Face-to-face; Helper-guided (multidisciplinary HCPs). Duration: 34 months; Multiple sessions of psychological counseling (≥ 5 times).	Psychosocial outcomes: not measured. TPT initiation (health facility medical records): Increased from 39–88%. RD: 49%; *p* < 0.001. Uptake of rapid MDR-TB testing: Increased from 67–92%; *p* < 0.001. RD: 25%. TB household contact screening: Increased from 82–96%; *p* < 0.02. RD: 14%. TPT completion rate (health facility medical records): Increased from 27–87%; *p* < 0.001. RD: 60%.	Moderate
2.	Alvarez et al.^39^	Settings: Community, Nunavut, Canada (HIC, LTB). Participants: Residents from six TB-high-incidence neighborhoods.	Study design: Uncontrolled pre-post design. Control group: Pre-intervention data from the standard public health programme.	Interventions: Multifaceted, community-based intervention comprising: a. Community-wide TB awareness campaign. b. Location-based door-to-door campaign: home education and LTBI screening (TST or IGRA). c. Home LTBI treatment. Education content: TB transmission, LTBI awareness, diagnosis, and preventive treatment. The material was culturally tailored for the Inuit population to reduce TB-related stigma and reinforce treatment motivation.	Audiovisual; Digital media and face-to-face; Helper-guided (multidisciplinary HCPs and CHWs). Duration: Multiple sessions; 26 months total: 5 months TB awareness, 6 months door-to-door screening, and 15 months treatment follow-up.	Psychosocial outcomes: not measured. Passive LTBI screening: Increased from an average of 26 to 50 people per month (*p* < 0,0002). TPT initiation (project records) was 61% in the intervention group versus 47% in the local program (RD: 14%). TPT completion (project records): 33% relative increase in the completion of TPT.	Moderate
3.	Wilson et al.^34^	Settings: Clinic and community, El Salvador (LMI, HTB). Participants: Household TB contacts and family members of individuals affected by TB.	Study design: Uncontrolled pre-post study. Control group: Pre-implementation clinic experience and baseline knowledge.	Interventions: A 7-minute Spanish educational video was shown on portable DVD players in the clinic waiting room and during household visits. Education content: a. TB definition, symptoms, transmission, diagnosis, treatment regimen, side effects, adherence, and common misconceptions. b. Testimonial from a former TB patient highlighting successful treatment and reintegration into daily life.	Audiovisual; Face-to-face; Helper-guided (multidisciplinary HCPs). Duration: 12 months; Multiple sessions (educational video was shown at least twice per patient).	Psychosocial outcomes: a. Treatment adherence: direct behavior observation; improvement in treatment adherence. b. Perceived social support: direct behavior observation; improvement in family support. c. TB knowledge: El Salvador Ministry of Health’s Questionnaire; improvement in knowledge of TB transmission (20–100%) (RD: 80%) and curability (13–100%) (RD: 87%).	Moderate
4.	Wingfield et al.^36^	Settings: Community, Peru (UMI, HTB). Participants: Household TB contacts aged < 20 years.	Study design: Household - randomized non-blinded controlled trial. Control group: Standard national TB Programme care (diagnosis, medication, and routine counseling).	Interventions: Social support: (1) health and financial education during home visits; (2) participation of individuals with TB and household contacts in monthly TB Clubs; (3) economic assistance, including conditional cash transfers of up to USD 230 per household. Contents: a. Education content: TB transmission, treatment regimen, and preventive therapy. b. Psychological support: TB Club to empower individuals through peer sharing experiences and reducing stigma. c. Financial education content: household finances management during TB care.	Verbal presentation; Face-to-face; Helper-guided (multidisciplinary HCPs). Duration: 16 months; Multiple sessions (TB clubs were held once a month, approximately six sessions).	Psychosocial outcomes: not measured. TPT initiation (health facility medical records): 43% in the intervention arm versus 25% in the control arm (aOR: 2,2; 95% CI: 1,1 – 4,1). RD = 18%.	High
5.	Yuen et al.^37^	Settings: Community, Peru (UMI, HTB). Participants: Household TB contacts aged < 20 years.	Study design: Uncontrolled pre-post study. Control group: Retrospective chart review of the baseline period (March–August 2015).	Interventions: Integrated socioeconomic activities include: (1) home visits to promote completion of evaluations; (2) transportation vouchers for access to health facilities (up to $5 for a round trip); (3) coordinating appointments for TST, chest X-ray, and pediatric TB consultations; (4) adherence counseling; and (5) monitoring for adverse events. Contents: a. Education content: Not specified. b. Psychological support: Home visits for encouragement, adherence counseling, and building trust with families.	Face-to-face; Helper-guided (multidisciplinary HCPs and CHWs). Duration: 6 – 7 months; Multiple sessions.	Psychosocial outcomes: not measured. LTBI evaluation completion (health facility medical records): Increased from 42–71% (RR = 1,73; 95% CI: 1,41 – 2,13). RD: 29%. TPT initiation (health facility medical records): Increased from 15–40% (RR: 2,45; 95% CI: 1,42 – 4,22). RD = 25%.	High
6.	Spruijt et al.^40^	Settings: Community, Netherlands (HIC, LTB). Participants: Eritrean migrants living in the Netherlands for < 10 years.	Study design: Mixed-methods implementation study. Control group: None.	Interventions: A package of activities for each strategy includes: (1) a 60 min educational multiple-session, (2) on-site QFT-Plus screening or scheduled appointments at a clinic, and (3) the provision of a 3-month HR treatment regimen for eligible participants. Education content: Short films highlighting the TB patient experience in diagnosis and treatment. The material was customized to align with Eritrean traditions, aiming to reduce stigma and skepticism.	Audiovisual and printed material; Face-to-face; Helper-guided (multidisciplinary HCPs and CHWs). Duration: Multiple sessions (10 sessions).	Psychosocial outcomes: Not measured. TPT initiation (project records): 97%, higher than observed among TB contacts in the Netherlands (52%). RD = 45% (project records). TPT completion rate (project records): 98%.	Moderate
7.	Hirsch-Moverman et al.^33^	Settings: Clinic and community, Lesotho (LMI, HTB). Participants: Caregiver of household TB child contacts.	Study design: Cluster-randomized controlled trial. Control group: Standard National TB Programme care: symptom screening, 6H if eligible, passive inconsistent follow-up, and limited CHWs involvement.	Interventions: Multicomponent community-based interventions include: (1) building capacity for nurses; (2) educating caregivers about LTBI in the waiting area; (3) conducting home visits for LTBI screening, TPT initiation, and weekly phone/SMS reminders for adherence. Education content: the importance of TB preventive treatment and treatment adherence.	Printed and verbal presentation; Face-to-face; Helper-guided (Multidisciplinary HCPs and CHWs). Duration: Up to 6 months per child; Multiple sessions.	Psychosocial outcomes: Not measured. TPT initiation (health facility medical records): 98% in the intervention arm versus 88% in the control arm (*p* < 0,0001). RD = 10% (the medical records of the health facility). TPT completion (direct observations): 82% in the intervention arm versus 59% in the control arm (p: 0,048). RD: 23%.	High
8.	Jerene et al.^32^	Settings: Community, Ethiopia (LMI, HTB). Participants: Caregiver of people with LTBI under 15.	Study design: Comparative pre-post study with concurrent controls. Control group: Ethiopian National TB programme (no Iddir involvement, contact investigation by CHWs is rarely performed).	Interventions: a. Iddir volunteers reached out by receiving index-case lists, visiting homes, screening symptoms, giving referral slips, verifying health center linkages, and doing daily or weekly follow-ups. b. Adherence support: regular in-person visits plus daily SMS and phone reminders. Contents: a. Education content: The article did not provide details on the educational content; it only mentioned that the education was designed to enhance caregivers’ understanding of TPT benefits. b. Psychological support: Iddir provided emotional and social support to caregivers as part of their follow-up role during TPT care.	Verbal; Face-to-face; Helper-guided (CHWs). Duration: 12 months; Multiple sessions.	Psychosocial outcomes: Not measured. TPT initiation (health facility medical records): Improvement in TPT uptake among children under 15 years, from 28.7–63.5% in the intervention group, and from 34.6–43.2% in the control group (*p* < 0.001). RD = 20,3%. TPT completion (health facility medical records): 99% completed treatment.	Moderate
9.	Magumba et al.^38^	Settings: Clinic, Namibia (UMI, HTB). Participants: PLHIV backlog cohort on ART (before July 31, 2018). New cohort (initiated ART after August 1, 2018).	Study design: Uncontrolled pre-post study. Control group: Baseline TPT coverage as the comparator.	Interventions: The intervention included: (1) daily health education sessions on TPT for all clinic clients; (2) printed materials (leaflets/flyers) distributed to clients. Education content: TB prevention regimen therapy, eligibility criteria, treatment duration, and potential side effects.	Printed and verbal presentation; Face-to-face; Helper-guided (Multidisciplinary HCPs). Duration: 2 years with daily education (± 15 minutes); Multiple sessions.	Psychosocial outcomes: Not measured. TPT initiation (health facility medical records): Increased TPT uptake in backlog (42–94%) and new cohort (81–100%). RD backlog cohort: 52%, and new cohort: 19%.	Low

*6 H* 6-month isoniazid, *ART* antiretroviral therapy, *DOPT* directly observe preventive therapy, *HR* isoniazid and rifampicin, *IGRA* interferon-gamma release assay, *QFT-Plus* quantiFERON-TB gold plus, *TST* tuberculin skin test.

### Models of psychosocial intervention

All studies included in this review involved various psychosocial interventions, which were categorized into four categories: (1) health education only^34,38^; (2) material support along with psychological counseling, home visits, and community workshops^35^; (3) material support combined with community-based support and home visits^36,37^; and (4) household-based outreach integrated with health education sessions^32,33,39,40^. For intervention targets, seven studies (77.8%) targeted people with LTBI, such as household contacts of TB patients^32–37^ and PLHIV^38^. Two studies (22.2%) focused on broader community groups: one targeted residents in areas with high TB incidence rates, while the other specifically addressed high-risk migrant populations^39,40^.

These psychosocial models were delivered via a variety of intervention formats. Community-based delivery was the predominant approach (66.7%), highlighting the importance of reaching participants directly within their living environments^33,35–37,39,40^. Magumba et al.^38^ focused solely on interventions in clinical settings, whereas two studies (22.2%) employed a combined approach that integrated clinical interventions with household outreach, helping to reach more family members and foster greater community engagement^33,34^.

The educational content of these psychosocial interventions covered a variety of essential topics related to TB. The most frequently addressed themes included the following: benefit, regimens, duration of TPT (88.8%)^32–36,38–40^, TB transmission (33.3%)^34,36,39^, TB symptoms and LTBI awareness (*n* = 2, 22.2%)^34,39^, treatment adherence (33.3%)^33–35^, potential side effects (22.2%)^34,38^, LTBI common misconceptions (22.2%)^34,39^, and survivor testimonials to motivate participants (44.4%)^34–36,40^. Rocha et al.^35^ and Wingfield et al.^36^ enhanced educational support by providing financial management during TB care. Additionally, Rocha et al.^35^ empowered communities through mobilization workshops that offered training on how to register for health insurance and guidance on income-generating skills. Educational materials were presented in audiovisual formats (33.3%)^34,39,40^, printed materials (22.2%)^33,38^, and verbal presentations during workshops and home visit descriptions (44.5%)^32,35–37^.

In addition to health education, six studies (66.6%) provided targeted psychological and social support, which included structured counseling for managing depression^35^, strategies to reduce community TB-related stigma^34–36,40^, and emotional support to encourage treatment adherence^32,37^. Efforts to reduce stigma have been made through various methods, such as short films featuring local community figures^39,40^, testimonies from TB survivors in regional languages to increase community engagement^34,39^ and peer support^35,36^. All the included articles (100%) utilized multisession methods over several months, promoting ongoing engagement and supporting the successful initiation and completion of TPT. The longest intervention lasted 34 months, although detailed information regarding the specific number or duration of sessions was reported inconsistently across the studies.

### Intervention providers

The psychosocial interventions in four studies (44.4%) involved helper-guided approaches featuring either multidisciplinary healthcare professionals alone^34–36,38^ or a combined model of multidisciplinary healthcare professionals and community health workers (55.6%)^32,33,37,39,40^. The multidisciplinary healthcare teams, which include physicians, psychologists, nutritionists, and TB nurses, work primarily in clinical or structured outreach settings. Their duties included clinical examination, diagnosis, monitoring of adverse events, providing nutritional and financial support, and training CHWs. The CHWs included health extension workers (HEWs), village health workers, women-led community associations, and community health workers linked with NGOs. They were responsible for conducting household visits, providing peer education, mobilizing the community, screening for symptoms, offering psychological and emotional support, reducing stigma, facilitating healthcare referrals, and delivering ongoing support to encourage treatment adherence.

### Quality of the included studies

The total CCAT scores ranged from 37.5–85% (Supplementary Material Table 2). Three studies were classified as high quality^33,36,37^, five as moderate quality^32,34,35,39,40^ and one as low quality^38^. The studies indicated that the preliminaries had the highest average score of 5.0, followed by the introduction at 4.78. This reflects consistent clarity in framing, structured abstracts, and well-articulated rationales for the studies. In contrast, the most frequently underperforming domains were design (2.33), sampling (2.78), and data collection (2.78), highlighting methodological weaknesses.

The low average score in the design domain stemmed from a lack of control groups in six studies (67%), which limited causal inference^34,35,37–40^. Additionally, four studies (44%) did not report whether concurrent changes in the National Tuberculosis Programs (NTPs) had any influence on outcomes. Additionally, three studies (33.0%) failed to provide details on the educational content offered. They did not clarify the intensity or frequency of psychosocial counseling or home visits, which are essential for assessing implementation fidelity. None of the studies included reported on sample size calculations or provided statistical justification for their sampling methods. This weakness undermines the assessment of whether the study was sufficiently powered to detect meaningful differences in outcomes.

In the data collection domain, two studies relied only on project-based records, which are more prone to measurement bias^39,40^. In contrast, seven studies utilized routine health facilities or national database systems^32–38^. Five studies reported treatment completion rates; four studies^32,35,39,40^ relied solely on medical or project records, which may have overestimated adherence. Only one study used the pill count method, which offers a more objective measure of treatment adherence and reduces the risk of overreporting^33^.

### Outcome and challenges of psychosocial interventions

The majority of the included studies (75.0%) relied on quantitative assessments derived from health facility medical records^32,33,35–38^ and project records^39,40^. These data sources were used to evaluate TPT uptake and, to a limited extent, psychosocial outcomes. Although both quantitative and qualitative approaches were used across the studies, there were considerable methodological limitations. Wilson et al. employed a questionnaire that included six basic items measuring TB knowledge. However, no information was provided regarding the validation of this questionnaire^34^. Similarly, Rocha et al. assessed stigma via an instrument lacking validation information^35^.

Importantly, not all studies have conducted statistical hypothesis testing to support their findings on the initiation of TPT. The corresponding RD for TPT initiation across all included studies are also summarized in Table 1 to ensure the comparability of outcomes. Among the studies reviewed, the majority (55.6%) included studies that provided quantitative estimates, such as risk ratios, odds ratios, or p values^32,33,35–37^. In contrast, four studies (44.4%) reported only descriptive results without conducting significance tests^34,38–40^. This inconsistency undermines the validity of the conclusions regarding the intervention's effects. Moreover, the assessment of psychosocial outcomes was limited, with only one study directly measuring psychosocial determinants as primary outcomes^34^. This intervention resulted in improvements in TB knowledge, perceived family support, treatment adherence, and a decrease in TB-related stigma, as assessed through both quantitative questionnaires and direct observations^34^.

All included studies reported improvements in TPT initiation following the implementation of psychosocial interventions. The risk difference ranged from 10–52%, depending on the study design and intervention model. Rocha et al. and Spruijt et al. utilized multicomponent interventions, with high RD rates of 49 and 45%, respectively^35,40^. Magumba et al. reported the greatest increase in TPT initiation, with a 52% risk difference within the backlog cohort^38^. Several studies have identified challenges that could hinder the scalability of psychosocial interventions. Technical issues, such as poor audio-visual quality and equipment malfunctions, impede the delivery of health education interventions that rely on audio-visual formats^34,38^. The provider considered the household-based outreach intervention to be time-consuming, which raised concerns about its feasibility in standard healthcare practices^40^. Scheduling household outreach during working hours made participation challenging^37^. Moreover, geographical and environmental factors complicate implementation efforts in some areas^39^.

## Discussion

This systematic review synthesizes evidence evaluating psychosocial interventions to increase TPT initiation among people with LTBI. All included studies incorporated education as a core component, underscoring its vital role in fostering patient awareness and motivation. The interventions varied from standalone education to an integrated model that combines economic support, psychological counseling, household-based outreach, and community mobilization.

This review identified several studies that included combined interventions such as socioeconomic and psychological support. However, none have been specifically designed to distinguish the unique effects of each component. Consequently, it remains unclear whether one form of support is more effective than the other in improving TPT uptake^36,37^. Nevertheless, multicomponent interventions have successfully targeted a broader range of psychosocial determinants simultaneously, including stigma, perceived risk-benefit, family support, and material barriers^9,11,41,42^. This suggests that combining elements may yield synergistic effects by addressing the complex and interconnected factors that influence individuals' decisions to initiate preventive therapy.

**Fig. 2:**
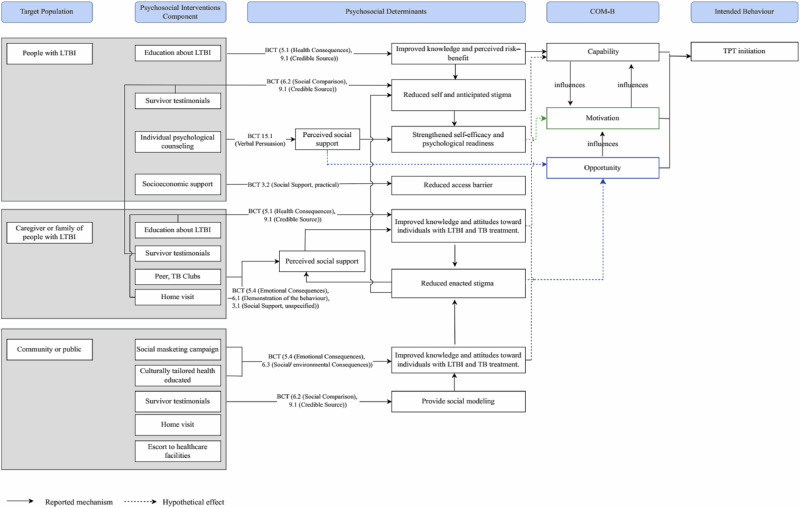
Conceptual mapping of psychosocial intervention, determinants, and behavior change pathways influencing TPT initiation.

All included studies reported improvements in TPT initiation. To explore the underlying mechanisms, we applied a COM-B and BCT-aligned framework. This review coded psychosocial intervention components with the BCT taxonomy version 1^30^ and linked them to psychosocial determinants as mechanistic pathways of behavior change. These pathways were subsequently mapped to the relevant COM-B domains (Fig. 2). However, only one of the included studies directly measured psychosocial determinants^34^. As a result, most pathways remain theory-informed and require further empirical validation. This gap underscores the pressing need for future research to measure psychosocial constructs and validate the mechanisms linking psychosocial interventions to the initiation of TPT.

The mapping illustrates multiple complementary pathways to promote TPT initiation. BCT 5.1 (Health Consequences) and BCT 9.1 (Credible Source) were the most common strategies, typically delivered through health education and survivor testimonials. These techniques enhanced psychological capability via treatment literacy while simultaneously reinforcing reflective motivation by highlighting the benefits of TPT^9,40,43^. Psychoeducation in particular demonstrated strong potential to influence individual decision-making. Rather than merely conveying information, psychoeducation catalyzes both attitudinal and behavioral change^44–46^. BCT 6.2 (Social Comparison) was also featured prominently. Survivor testimonials, peer groups, and TB clubs allowed individuals to compare themselves with others. These methods reduced anticipated stigma and enhanced social opportunities. Other strategies include BCT 5.4 (Emotional Consequences), BCT 6.1 (Demonstration of Behavior), and BCT 3.1 (Social Support, unspecified). These approaches provided emotional reinforcement, behavioral modeling, and peer encouragement. As a result, these strategies can diminish self-stigma and promote psychological readiness, acting mainly through the domains of motivation and opportunity^27,36,43^.

Structural and psychological support further strengthened the initiation of TPT. BCT 3.2 (Social Support, practical) addressed structural barriers through socioeconomic support, home visits, and accompaniment to health facilities, thereby enhancing physical opportunity^37,44^. Similarly, BCT 15.1 (Verbal Persuasion about Capability) and BCT 6.3 (Social/Environmental Consequences) contributed to strengthening self-efficacy, altering perceptions of stigma, reframing LTBI as a manageable condition, and making the treatment journey more relatable^34,39,40^. These strategies illustrate how capability-building and opportunity-enhancing approaches act synergistically with motivation-strengthening components. Taken together, these findings indicate that psychosocial interventions do not operate through a single mechanism. Instead, they rely on a constellation of BCTs that work across COM-B domains. This multicomponent approach underscores the importance of psychosocial interventions that are both theory-informed and contextually tailored to maximize their impact on TPT initiation.

Interventions in the included studies were carried out primarily in community-based settings, highlighting their ability to engage target populations directly within their social environments. Notably, in LMI contexts such as Lesotho, psychosocial interventions were successfully delivered across both clinical and community settings^33^. These dual-setting interventions demonstrated significant acceptability, even in health systems with limited formal workforce capacity, due to the active participation of CHWs and prior capacity-building efforts^33^. This finding suggests that with appropriate support systems, it is possible to effectively integrate clinic- and community-based outreach, even in resource-limited settings, to promote behavior change and enhance TPT uptake. A common feature across all interventions was their delivery by helper-guided, primarily multidisciplinary HCPs and CHWs. This interdisciplinary model reflects the reality that decisions regarding TPT initiation are influenced not only by clinical information but also by psychological readiness, social support, and contextual barriers^9,16^. However, none of the included studies reported the involvement of pharmacy personnel, even though their role in TPT adherence and decision-making is well recognized^14,47^. Evidence from collaborative LTBI care models in the United States demonstrates that pharmacists not only improved treatment completion rates but also reduced workload for public health departments^48,49^. Their expertise aligns closely with psychosocial determinants in the COM-B framework. Pharmacists can strengthen their psychological ability by enhancing treatment literacy, reinforcing reflective motivation through counseling that addresses stigma and concerns about side effects, and expanding opportunities by offering accessible medication-related support in community settings^48–50^. The lack of pharmacy engagement in these efforts highlights a missed opportunity, underscoring the need for future programs to integrate pharmaceutical services into psychosocial strategies, thereby ensuring that both the psychological and pharmacological aspects of TPT are appropriately addressed.

CHWs play a vital role in expanding program reach, reducing the strain on HCPs, and ensuring consistent service delivery across dispersed populations^32,51^. The involvement of TB champions, community leaders, and volunteers further enhanced the credibility and cultural relevance of the interventions. Their ability to leverage local trust helps address sociocultural and health system barriers to treatment acceptance^40^. Notably, two studies have adopted participatory or cocreation approaches in the design and delivery of interventions. This approach fosters key components for sustainable interventions, such as a sense of ownership, contextual relevance, and community engagement^18,39,40,52^. Audiovisual formats often deliver standardized, engaging, and culturally relevant messages, particularly when accompanied by guided assistance. Interventions were typically conducted over several sessions across multiple months, allowing time for reinforcement, relationship-building, and adaptation. This continuity is crucial for promoting behavioral changes in preventive therapies, which often require anticipatory decision-making in the absence of immediate clinical symptoms^43^.

Despite promising outcomes, a significant limitation of the existing evidence base is the absence of robust statistical analyses and study designs in numerous studies. Approximately 44.4% of the included articles reported descriptive improvements in TPT initiation without performing hypothesis testing or presenting measures of statistical significance. In contrast, studies with more rigorous designs, such as randomized controlled trials and controlled pre-post observations, reported relatively smaller effect sizes than those with uncontrolled pre-post designs^32,33,36^. This discrepancy highlights the importance of cautious interpretation, particularly in studies lacking control groups, which are more susceptible to confounding influences. It is also essential to consider that the included studies employed a variety of study designs, ranging from uncontrolled pre-post studies to RCTs. This heterogeneity in study design and methodological rigor poses a limitation in performing a meta-analytic study and comparing effect sizes across studies. This may influence the strength of the conclusions that can be drawn about the relative effectiveness of each intervention model.

In addition to the study design, another key limitation is the insufficient reporting of psychosocial interventions. Many studies fail to provide details on the frequency, duration, or fidelity of intervention delivery. This lack of information limits the assessment of intervention intensity and hinders replication across settings. Psychosocial constructs, such as stigma and perceived social support, were assessed using instruments that lacked clear evidence of validation. Reliance on unvalidated tools reduces confidence in the reported effects and complicates cross-study comparisons. To address these gaps, future research should adapt standardized reporting frameworks, such as the Template for Intervention Description and Replication (TIDieR) checklist, and consistently employ validated psychosocial measurement tools^53^. Such practices would enhance transparency, improve comparability, and strengthen the reliability of evidence for both researchers and implementers.

The geographical distribution of studies also warrants consideration. Most existing evidence comes from upper-middle-income countries with a high burden of TB. However, there is a notable absence of research from Asia, despite the region's substantial burden of LTBI and consistently low rates of TPT initiation. High-burden TB countries in Asia, such as India, Indonesia, China, and the Philippines, are critical areas where customized psychosocial interventions are urgently needed^1^. This gap largely reflects prevailing programmatic and research priorities. National TB programs in many Asian countries remain focused primarily on the urgent demands of TB disease control, whereas LTBI management is treated as secondary due to its asymptomatic nature^54^. Bibliometric analyses confirmed that between 1995 and 2018, research output from high-burden countries concentrated on TB disease. In contrast, studies from low-burden settings gradually shifted their attention to LTBI as TB disease became better controlled^55^. At that time, efforts to scale up preventive therapy in Asia were further constrained by logistical and pharmaceutical barriers, including limited access to rifapentine and child-friendly formulations^56^. Under such conditions, psychosocial interventions are unlikely to be prioritized when the biomedical foundations of LTBI management remain insufficiently established. This gap underscores the need for developing regionally tailored psychosocial research to enhance TPT uptake in high-burden Asia settings.

### Strengths and limitations

This review significantly contributes to the growing body of evidence on psychosocial interventions aimed at increasing TPT initiation by identifying various components, highlighting effective strategies, and contextualizing adaptations across different settings. To our knowledge, this is the first review to focus on psychosocial interventions targeting the LTBI population, integrating data on TPT initiation and psychosocial factors. Nevertheless, several limitations should be acknowledged. First, we were unable to perform a meta-analysis due to heterogeneity in interventions, outcomes, populations, and study designs. Instead, we employed narrative synthesis, which may be a more subjective approach. Second, the methodological quality of the included studies was generally moderate to low, with many employing quasi-experimental or observational methods and limited adjustments for confounders. Third, the direct measurement of psychosocial determinants was limited, leaving the mechanism through which interventions may improve TPT initiation largely hypothetical. This limitation restricts causal interpretation and underscores the need for more rigorous research. Fourth, details on intervention delivery (e.g., frequency, duration, fidelity) and the use of validated psychosocial measures were often insufficient, which constrains comparability and replication. Despite these concerns, this review offers valuable insights for future research, policy, and practice.

## Conclusion

This review affirms the potential of community-driven, theory-informed psychosocial interventions in supporting TPT initiation among people with LTBI. Impactful interventions tend to integrate education with social and psychological support, leverage community-based delivery, and engage trusted local actors. However, the strength of the current evidence base is tempered by low to moderate methodological quality, significant geographic gaps, limited use of validated tools and outcomes for psychosocial aspects. Multicomponent, community-based approaches delivered by multidisciplinary healthcare professionals and community health workers, incorporating culturally tailored health education along with survivor testimonials, appear particularly well-suited to address the complex psychosocial barriers that influence treatment decisions. Future studies should prioritize high-quality study designs, rigorous outcome evaluations that include psychosocial determinants, and a greater focus on implementation in high-burden TB settings in Asia. These efforts will be crucial in informing scalable psychosocial strategies and advancing progress toward the global goal of TB elimination.

## Supplementary information

Supplementary Material Table 1 and 2

## Data Availability

No datasets were generated or analysed during the current study.
