# Supplementary material for: Psychosocial interventions to improve tuberculosis preventive treatment uptake and psychosocial outcomes: a systematic review
Source: NPJ Prim Care Respir Med. 2025 Sep 29;35:40. doi: 10.1038/s41533-025-00449-3 (PMC12480859; doi:10.1038/s41533-025-00449-3)
Supplement: Supplementary file 1 — Supplementary Material Table 1 and 2 [file 41533_2025_449_MOESM1_ESM.docx]

**Supplementary Material**

**Table 1.** Searching Strategy

| **Scopus (March 12, 2025)** | | |
| --- | --- | --- |
| **Population** | | ( TITLE-ABS-KEY ( latent AND tuberculosis ) OR TITLE-ABS-KEY ( latent AND tb ) OR TITLE-ABS-KEY ( tuberculosis AND infection ) OR TITLE-ABS-KEY ( ltbi AND latent AND tb AND infection ) OR TITLE-ABS-KEY ( hiv AND aids ) OR TITLE-ABS-KEY ( hiv ) OR TITLE-ABS-KEY ( household AND contact* ) ) |
| **Intervention** | | ( TITLE-ABS-KEY ( psychosocial AND intervention ) OR TITLE-ABS-KEY ( psychosocial AND care ) OR TITLE-ABS-KEY ( counseling ) OR TITLE-ABS-KEY ( education ) OR TITLE-ABS-KEY ( nurse-led AND intervention* ) OR TITLE-ABS-KEY ( community-based AND intervention ) OR TITLE-ABS-KEY ( person-centered AND approach ) OR TITLE-ABS-KEY ( pharmacist AND led AND intervention ) OR TITLE-ABS-KEY ( campaign* ) OR TITLE-ABS-KEY ( psycholg* AND intervention ) OR TITLE-ABS-KEY ( motivational AND interviewing ) OR TITLE-ABS-KEY ( cognitive AND behavioral AND therapy ) OR TITLE-ABS-KEY ( cbt ) ) |
| **Outcome** | | ( TITLE-ABS-KEY ( tuberculosis AND prevent* AND therapy ) OR TITLE-ABS-KEY ( tuberculosis AND prevent* AND treatment ) OR TITLE-ABS-KEY ( tpt ) OR TITLE-ABS-KEY ( tpt AND uptake ) OR TITLE-ABS-KEY ( 3hp ) OR TITLE-ABS-KEY ( isoniazid* AND uptake ) ) |
| **Combination** | | ( TITLE-ABS-KEY ( latent AND tuberculosis ) OR TITLE-ABS-KEY ( latent AND tb ) OR TITLE-ABS-KEY ( tuberculosis AND infection ) OR TITLE-ABS-KEY ( ltbi AND latent AND tb AND infection ) OR TITLE-ABS-KEY ( hiv AND aids ) OR TITLE-ABS-KEY ( hiv ) OR TITLE-ABS-KEY ( household AND contact* ) ) AND ( TITLE-ABS-KEY ( psychosocial AND intervention ) OR TITLE-ABS-KEY ( psychosocial AND care ) OR TITLE-ABS-KEY ( counseling ) OR TITLE-ABS-KEY ( education ) OR TITLE-ABS-KEY ( nurse-led AND intervention* ) OR TITLE-ABS-KEY ( community-based AND intervention ) OR TITLE-ABS-KEY ( person-centered AND approach ) OR TITLE-ABS-KEY ( pharmacist AND led AND intervention ) OR TITLE-ABS-KEY ( campaign* ) OR TITLE-ABS-KEY ( psycholg* AND intervention ) OR TITLE-ABS-KEY ( motivational AND interviewing ) OR TITLE-ABS-KEY ( cognitive AND behavioral AND therapy ) OR TITLE-ABS-KEY ( cbt ) ) AND ( TITLE-ABS-KEY ( tuberculosis AND prevent* AND therapy ) OR TITLE-ABS-KEY ( tuberculosis AND prevent* AND treatment ) OR TITLE-ABS-KEY ( tpt ) OR TITLE-ABS-KEY ( tpt AND uptake ) OR TITLE-ABS-KEY ( 3hp ) OR TITLE-ABS-KEY ( isoniazid* AND uptake ) ) |
| **Results** | | 1.082 articles |
| **Pubmed (March 21, 2025)** | | |
| P1 | (("Tuberculosis/prevention and control"[Mesh]) OR ( "Latent Tuberculosis/drug therapy"[Mesh] OR "Latent Tuberculosis/psychology"[Mesh] )) | |
| P2 | "AIDS-Related Opportunistic Infections/prevention and control"[Mesh] | |
| P3 | "Household contact" [Title/Abstract] | |
| P4 | “HIV” [Title/Abstract] OR “TB” [Title/Abstract] OR “Latent Tuberculosis” [Title/Abstract] OR “Latent TB” [Title/Abstract] OR "Household contact" [Title/Abstract] | |
| I1 | (("Psychosocial Intervention"[Mesh]) OR "Health Education"[Mesh]) OR "Counseling"[Mesh] | |
| I2 | “Psychosocial Intervention” [Title/Abstract] OR “Counsel*” [Title/Abstract] OR “Lay Worker” [Title/Abstract] “Psychology* Intervention” [Title/Abstract] OR “Health Education” [Title/Abstract] OR “Education” [Title/Abstract] OR “Campaign” [Title/Abstract] OR “ Voucher” [Title/Abstract] OR “Transport*” [Title/Abstract] OR “Incentive*” [Title/Abstract] OR “Community based intervention”[Title/Abstract] OR “Motivational interviewing” [Title/Abstract] OR “acceptance and commitment” [Title/Abstract] OR “cognitive-behavioral therapy” [Title/Abstract] OR “CBT” [Title/Abstract] | |
| I3 | “Patient-centered care” [Title/Abstract] OR “patient-centred care” [Title/Abstract] OR “people-centered care” [Title/Abstract] OR “people-centred care” [Title/Abstract] | |
| O1 | (("Antitubercular Agents/therapeutic use"[Mesh]) OR "Isoniazid/therapeutic use"[Mesh]) OR "rifapentine" [Supplementary Concept] | |
| O2 | "Tuberculosis Prevent* Therapy" [Title/Abstract] OR "Tuberculosis Prevent* Treatment"[Title/Abstract] OR "TPT Uptake" [Title/Abstract] OR "TPT Initiation" [Title/Abstract] OR "Isoniazid Uptake" [Title/Abstract] OR "Isoniazid Initiation" [Title/Abstract] | |
| 1 | P1 OR P2 OR P3 OR P4 | |
| 2 | I1 OR I2 OR I3 | |
| 3 | O1 OR O2 | |
| 1 AND 2 AND 3  **Results:** 704 articles | | |
| **PsycInfo (August 25, 2025)** | | |
| P | Index Terms: {Pulmonary TUBERCULOSIS} OR {TUBERCULOSIS} | |
| I | Index Terms: {Support Groups} OR {Psychosocial Rehabilitation} OR {Psychosocial Outcomes} OR {Psychosocial Factors} OR {Psychoeducation} OR {Cognitive Stimulation Therapy} OR {Psychosocial Interventions} OR {Psychoeducation} OR {Health Education} OR {Education} AND | |
| O | Index Terms: {Treatment Compliance} | |
| **Results** | 55 | |

**Table 2.** CCAT score

| **Author** | **Preliminaries** | **Introduction** | **Design** | **Sampling** | **Data Collection** | **Ethical matters** | **Results** | **Discussion** | **Total** | **Total (%)** | **Low/ moderate/ high quality** |
| --- | --- | --- | --- | --- | --- | --- | --- | --- | --- | --- | --- |
| Rocha et al., 2011 | 5 | 5 | 3 | 3 | 3 | 2 | 3 | 4 | 28 | 70 | Moderate |
| Alvarez et al., 2014 | 5 | 5 | 3 | 2 | 2 | 5 | 3 | 3 | 28 | 70 | Moderate |
| Yuen et al., 2019 | 5 | 5 | 3 | 3 | 4 | 5 | 3 | 3 | 31 | 77.5 | High |
| Spruijt et al., 2020 | 5 | 5 | 1 | 3 | 3 | 5 | 3 | 4 | 29 | 72.5 | Moderate |
| Wilson et al., 2016 | 5 | 5 | 1 | 3 | 2 | 3 | 2 | 3 | 24 | 60 | Moderate |
| Wingfield et al., 2017 | 5 | 5 | 4 | 4 | 3 | 5 | 4 | 4 | 34 | 85 | High |
| Hirsch-Moverman et al., 2022 | 5 | 5 | 2 | 3 | 4 | 5 | 4 | 4 | 32 | 80 | High |
| Jerene et al., 2022 | 5 | 5 | 3 | 2 | 3 | 4 | 4 | 3 | 29 | 72.5 | Moderate |
| Magumba et al., 2023 | 5 | 3 | 1 | 2 | 1 | 0 | 1 | 2 | 15 | 37.5 | Low |
| **Average Score** | 5.00 | 4.78 | 2.33 | 2.78 | 2.78 | 3.78 | 3.00 | 3.33 |  |  |  |
